# Calendar

**DOI:** 10.1155/S146392468000017X

**Published:** 1980


					( alenclar
Editor's Note:
Organisers of conferences, seminars etc.
should send details for inclusion in this
calandar as soon as the relevant informa-
tion is available and not later than three
months before the event.
Sampling
January 18, Chepstow, Wales
The discipline of chemical education
research
February 28, London
Stanley Langer, The Chemical Society,
Burlington, London W1 V OBN, UK.
Pigment analysis in the scientific exami-
nation of paintings in the National
Gallery
March 6, London
The ScientificLecture Organiser, Labora-
tory of the Government Chemist,
Cornwall House, Stamford Street,
Miss P. E. Hutchinson, The Chemical London SE1 9NQ, UK.
Society, Analytical Division, Burlington
House, London W1 V OBN, UK.
Analyst- on and off the rails
January 18, Salford, UK.
Mlss P. E. Hutchinson, The Chemical
Society, Analytical Division, Burlington
House, London W1 V OBN, UK.
The Pittsburgh Conference
March 10-14, Atlantic City, USA
Dr R S Danchik, 25 Thorncrest Drive,
Pittsburgh, PA 15235, USA
International Chromatography Con-
ference
March 20-21, Florida
Practical immunoassay-thepresentstate Alena Enterprises of Canada, PO Box
of the art 1779, Cornwall Ontario, Canada K6H
January 24, Birmingham
Miss P. E. Hutchinson, The Chemical
Society, Analytical Division, Burlington
House, London W1 V OBN, UK.
Recent advances in bio-organic chemistry
January 24, London, UK
Stanley Langer, The Chemical Society,
Burlington House, London W1V OBN,
UK.
5V77
7th Annual Report on Analytical Atomic
Spectroscopy Symposium
March 27, Sheffield.
D. J. Willis, LRIC, Rank Hilger, West-
wood Industrial Estate, Ramsgate Road,
Margate, Kent, UK.
Annual Chemistry Congress
April 9 11, Durham
Dr. J.F. Gibson, The Chemical Society,
Recent advances in organicmassspectro- Burlington House, London WI F OBN.
metry in analytical chemistry
February 6, London
Miss P. E. Hutchinson, The Chemical
Society, Analytical Division, Burlington
House, London W1 V OBN, UK.
Sophistication in instrumentation -has
it gone too far?
February 13, London
D. J. Willis, LRIC, Rank Hilger, West-
wood Industrial Estate, Ramsgate Road,
Margate, Kent, UK.
2nd International Congress on Phos-
phorous Compounds
April 21 25, Boston
IMPHOS, 8 rue de Pentievre, 75008,
Paris, France.
Labware '80
April 22-24, London
Labware Promotions, 28 Worple Road,
London SW19 4EE, UK.
Advances in applied high-performance
liquid chromatography
February 20, Edinburgh, UK
Diana Simpson, Analysis For Industry,
Factories 2/3, Bosworth House, High
Street, Thorpe-le-Soken, Essex C016
OEA, UK.
Programming microprocessors for
industrial measurement and control
April 29-30, London
Mrs R G Keiller, Sira Institute Ltd,
South Hill, Chislehurst, Kent BR 7
5EH, UK.
Analytiea '80
April 29 May 2, Munich
ECL (Exhibition Agencies) Ltd., 11
Manchester Square, London Wl
Phirama 80 scientific information and
technique exhibition
May 6-9, Marseilles
Foire de Marseilles, Service Phirama,
Parc Chanot, 13008 Marseilles, France.
Analytical chemistry of pollutants
May 28-30, Dortmund, FRG
Dr J Wendenburg, Gesellschaft Deut-
scher Chemiker, PO Box 900440,
D-6000 Frankfurt am Main 90, FRG
High speed automatic analysis
May 30, Glasgow
D. G. Porter, Laboratory of the Govern-
ment Chemist, Cornwall House,
Stamford St, London SE1 9NQ
Eurochem '80
June 23-27, Birmingham
Andrew Dedman, Clapp & Poliak Europe
Ltd, 232 Acton Lane, London W4 5DL,
UK.
Swansea summer school of Automatic
Chemical Analysis
July 6-11, Swansea
Dr J. Betteridge, University College of
Swansea, Singleton Park, Swansea, Wales.
SAC 80
July 20-26, Lancaster
The Secretary, Analytical Division,
Chemical Society, Burlington House,
London W1 V OBN, UK.
Laboratory '80
September 9-11, London
Curtis Steadman, 34-36 High Street,
Saffron Walden, Essex.
1981
Euroanalysis IV
August 23-28, Helsinki, Finland
Association of Finnish Chemical
Societies, Executive Secretary, Pohj,
Hesperiankatu 3B10, SF-oo260 Helsinki
26, Finland
Volume 2 No. January 1980 43

				

